# Who uses NHS health checks? Investigating the impact of ethnicity and gender and method of invitation on uptake of NHS health checks

**DOI:** 10.1186/s12939-016-0303-2

**Published:** 2016-01-20

**Authors:** Erica J. Cook, Chloe Sharp, Gurch Randhawa, Andy Guppy, Raj Gangotra, Jonathon Cox

**Affiliations:** Department of Psychology, University of Bedfordshire, Park Square, Luton, UK; Institute for Health Research, University of Bedfordshire, Putteridge Bury, Hitchin Road, Luton, UK; Public Health Department, Northamptonshire Council, Northampton, UK; Public Health Department, Norfolk County Council, Norfolk, UK

**Keywords:** NHS Health Check, Ethnicity, Gender, Invitation method

## Abstract

**Background:**

NHS Health Checks is a national risk assessment prevention programme for all individuals aged 40-74 that reside in England. Through the systematic assessment of an individual’s ten year disease risk, this programme aims to provide early identification and subsequent management of this risk. However, there is limited evidence on how socio-demographic factors impact on uptake and what influence the invitation method has on uptake to this programme.

**Methods:**

NHS Health Check data from April 2013 to March 2014 was analysed (*N* = 50,485) for all 30 GP Practices in Luton, a culturally diverse town in England, UK. Data was collected for age, ethnicity, uptake (attendance and non attendance) and invitation method (letter written, verbal face-to-face, telephone). Actual usage of NHS Health Checks was determined for each ethnic group of the population and compared using Chi-square analysis.

**Results:**

The overall uptake rate for Luton was 44 %, markedly lower that the set target of 50–75 %. The findings revealed a variation of uptake in relation to age, gender, level of deprivation. Ethnicity and gender variations were also found, with ‘White British’ ‘Black Caribbean’ and ‘Indian’ patients most likely to take up a NHS Health Check.

However, patients from ‘Any Other White Background’ and ‘Black African’ were significantly less likely to uptake an NHS Health Check compared to all other ethnic groups. Ethnicity and gender differences were also noted in relation to invitation method.

**Conclusions:**

The findings revealed that different invitation methods were effective for different ethnic and gender groups. Therefore, it is suggested that established protocols of invitation are specifically designed for maximizing the response rate for each population group. Future research should now focus on uncovering the barriers to uptake in particular culturally diverse population groups to determine how public health teams can better engage with these communities.

## Background

The NHS Health Check programme was formally launched in April 2009 as a population-wide disease prevention programme in England which aimed to improve life expectancy through the reduction of morbidity and mortality [1]. This programme is essentially a risk assessment which uses specific tests and measurements to systematically assess an individual’s ten year risk of developing heart disease, stroke, diabetes and kidney disease alongside raising a patients awareness of dementia [2, 3]. This provides an early identification of risk, which can then be used as a basis to inform a discussion with the patient surrounding lifestyle, and medical approaches that would be best suited to managing this risk. This preventative programme is aimed of the national population, targeting all individuals aged 40 to 74 years old who are not currently receiving treatment or support for any of the discussed conditions [3].

The Department of Health outlined that the implementation of this policy is likely to demonstrate national savings of £57 million over four years [2], however, the economic modelling of such savings remains dependent on a national uptake rate of 75 % of the 20 % eligible population receiving an NHS Health Check every five years [4]. Research has shown there is national variation in the uptake rate dependent on socio-demographic factors (ethnicity and gender) [5]. Research from a diverse setting with high levels of deprivation, highlighted a 44.8 % uptake rate varying by age, gender and ethnicity; with lower uptake found for younger men but higher uptake found for South Asian patients [6]. This is supported by more recent research which suggested increasing age, female status and living in an area with low levels of deprivation were all predictive of (positive) NHS Health Check uptake [7].

However, these findings have not been consistent. For example, research has suggested that NHS Health Checks were more likely to be completed by patients from ‘South Asian’ or ‘Mixed’ ethnic backgrounds compared to patients with their ‘White’ counterparts [6]. However, other research has suggested that non-Whites are still at more risk of developing cardiovascular disease and may not be benefiting from NHS Health Checks programme [5]. Therefore, there is a clear need to investigate the role of ethnicity in NHS Health Check uptake.

In response to the variation of uptake a recent review identified the need to understand the factors that influence the UK population response to invitations to attend the NHS Health Checks programme [8]. This is further supported by the recently published report by Public Health England ‘NHS Health Check Programme: Priorities for Research’ which clearly set out that evaluation of effective methods for inviting people to an NHS Health Check and evaluating equitable uptake of the programme are core priorities for research [2, 4]. To date, only one research study that has evaluated invitation method has been published [7]. This study found that there was a variation by method and geographical proximity whereby telephone/verbal invitations were associated with higher uptake compared to postal invitations, which varied by practice [7]. However, what is less clear is why this variation exists and if it could be explained by variation of ethnicity and gender as opposed to invitation method and geographical proximity variables.

The presented research has two key aims. Firstly, it aims to identify if there any systematic differences among socio-demographic differences in the uptake of NHS Health Checks in a culturally diverse town of England accounting for age, gender, deprivation and Secondly, this study aims to examine the methods of invitation to determine if some methods are more successful than others for specific population groups by ethnicity and gender.

## Methods

### Setting

The Department of Health aims for the NHS Health Check programme to be locally tailored to reach high-risk patients and those residing in hard-to-reach communities to engage in the programme to reduce health inequalities. The setting therefore for the present NHS Health Check evaluation is Luton, a culturally diverse, multi-ethnic and multi-faith town with a high rate of socioeconomic deprivation [9–11].

Luton is an ethnically diverse and aging town with a total population of 203,200 [12]. White British make up just under half of the population (47.7 %) with South Asians and Black African and Caribbean accounting for 26.4 % and 9.5 % of Luton’s total population respectively. There is also a high level of migration of European Union citizens from Poland and other Eastern European countries who account for 7 % of Luton’s total population [12]. In recent years, the diversity of the population has widened due to international students studying at the local university. Although National Insurance registrations have decreased recently, there is a significant rise in those registering from India and from Congo, Somalia, Ghana, Nigeria, Zimbabwe and Turkey [11].

In Luton, heart disease and stroke are higher than the national average [9]. Over a quarter of Luton’s population reside in quintile 1 ‘most deprived’ wards’ based on the Index for Multiple Deprivation [13]. It is suggested that males and females who reside in the most ‘deprived wards’ in Luton will have a lower life expectancy of 8.9 years and 6.4 years respectively compared to those in the least ‘deprived wards’ [9].

Public Health England set a national target for each area a target of 20 % of all eligible individuals to be offered an NHS Health Check with a target uptake rate of 50–75 % [9]. The eligibility criteria for an NHS Health Check refers to all individuals aged 40 to 74 years old. As this is a preventative programme anyone who is receiving treatment or support for coronary heart disease, chronic kidney disease (CKD) which has been classified as stage 3, 4 or 5 within, diabetes, hypertension, atrial fibrillation, transient ischaemic attack, hypercholesterolemia, heart failure, peripheral arterial disease, stroke are not eligible. Also anyone who has been prescribed statins, and during a previous NHS Health was found to have a 20 % or higher ten year risk of developing cardiovascular disease are also excluded [3].

### Dataset and sample

NHS Health Check data was extracted for all 30 GP practices in Luton, UK on behalf of the Luton Borough Council Public Health Department. All ‘patients’ 1 (*N* = 50,485) who were eligible for a NHS Health Check in Luton were included over the 12-month period from 1 April 2013 to 31 March 2014. This period was chosen for a number of reasons. Firstly, data completion was highest at this point as prior to this not all data was routinely entered. Secondly, this period was prior to the introduction of the free gym sessions to incentivise the uptake of an NHS Health Check, which could have biased the findings. Data was extracted directly from TCR (Nottingham), which is the responsible body for storing the GP practice NHS Health Checks data for Luton, UK [14].

Data extracted included patients who were eligible for a NHS Health check, patients who were offered an NHS Health Check and patients who have had an NHS Health Check. Demographic data (patient sex, age and ethnicity) were collected alongside NHS Health Check data. Age was recoded into 8 groupings (40–44; 45–49; 50–54; 55–59; 60–64; 65–69; 70–74). Ethnicity was categorised in line with the census 2001 [15] (see Table 1) as these were the same ethnic groupings that were coded by TCR (Nottingham). The Index of Multiple Deprivation (IMD) 2007 score was used as a measure of deprivation [16, 17]. All IMD measures were divided into five deprivation quintiles, with each quintile comprising of 20 % of the population of England. A higher IMD score or deprivation quintile indicated increasing deprivation.

**Table 1:** 2001 Census groupings for ethnicity

White	White: British
	White: Irish
	White: Other White
Mixed	Mixed: White and Black Caribbean
	Mixed: White and Black African
	Mixed: White and Asian
	Mixed: Other Mixed
Asian or Asian British	Asian: Indian
	Asian: Pakistani
	Asian: Bangladeshi
	Other Asian
Black or Black British	Black Caribbean
	Black African
	Other Black
Chinese or other ethnic group	Chinese
	Other

Invitation method was categorised by: (1) verbal (face-to-face) invitation (invited at GP practice); (2) contacted by telephone by the GP Practice and (3) written (sent an invitation letter from the GP practice to attend NHS Health Check). There is currently no standard protocol for recruitment although it remains the GP practice’s responsibility to invite eligible ‘patients’ to attend an NHS Health Check.

### Statistical analysis

The primary outcome measure was the uptake rate of the NHS Health Check programme during the 12-month period from 1 April 2013 to 31 March 2014. Uptake rate was calculated as eligible patients invited by the GP practice / those who had an NHS Health Check. Categorical variables for all explanatory variables were calculated. Chi-square Goodness of Fit analysis and Fishers Exact Tests were completed using weighted cases for frequency. Percentage uptake rates were calculated by dividing the absolute number of uptake by the total number of patients invited overall. Adjusted standardised residuals (ASR)’s were calculated to indicate the importance of the cell to the ultimate chi-square value which take account of the overall sample size. This was particularly important given the varying counts by uptake rate across groups. Therefore, when reporting the results, the ASR values were used to indicate significance i.e. ASR values of 3.09 (*p* < .001), 2.6 (*p* < .01) and 2 (*p* < .05) will signify significance, with anything below 2 deemed non-significant (*p* > .05). Binomial distribution was also used to calculate 95 % CIs for these rates. All statistical tests were completed using IBM SPSS Version 21, two-tailed significance was assumed at p < 0.05.

### Ethical approval

Luton Borough Council Public Health Department gave permission for the data to be used. The University of Bedfordshire Institute of Health Research Ethics Committee (REF: IHREC387; 13^th^ June 2014) and the Luton GP Caldicott Guardian provided ethical clearance. A data sharing protocol was set up by Luton Borough Council and the University of Bedfordshire. All data on the NHS Health Check database was anonymised and categorized prior data transfer to ensure no patient could be identified. GP practices were made anonymous before transfer and were allocated a pseudonym for the purposes of the statistical analysis.

## Results

The total number of patients aged 40–74 years who were eligible for an NHS Health Check during the 1^st^ April 2013 to 31^st^ March 2014 was 50,485 (Male: 26,372, Female: 24,113). In this period 13,063 patients were offered an NHS Health Check (Male: 6,962, Female: 6,101) with a total of 5,703 recorded as having an NHS Health Check. The overall uptake rate was 0.44 with a lower uptake rate shown for males (Male: 0.38, *p* < .001) when compared to females (0.50, *p* < .001).

### Socio-demographic factors and uptake of the NHS Health Check programme

#### Gender and level of deprivation

The deprivation quintile and gender of patients who were offered a Health Check was compared to actual uptake. Chi-square analysis revealed that uptake was not equally distributed across the total sample by ethnicity for both males (X^2^ = (4, *N* = 4222) = 70.979, *p* < .001) and (X^2^ = (4, *N* = 3703) = 99.427, *p* < .001).

The findings suggested that the significantly lowest uptake of the NHS Health Check was found from patients who resided from the most deprived wards (quintile 5) for both males and females with an uptake rate of 0.31 and 0.38 respectively (*p* < .001). In contrast, higher uptake of the NHS Health Check was found for patients who resided in the least deprived wards (quintile 1) with an uptake rate of 0.53 and 0.60 for males and females respectively (*p* < .001). The findings appear to suggest that patients who were invited to have an NHS Health Check who resided in the most deprived quintiles were less likely to have an NHS Health Check compared to patients within the least deprived quintiles.

### Age, gender and NHS Health Check uptake

The age and gender of patients who were offered a Health Check was compared to actual uptake. Chi-square analysis revealed that uptake was not equally distributed across the total sample by age for both males (X^2^ = 233.92, df = 7, *p <* .001) and females (X^2^ = 136.74, df = 7, *p <* .001) (Table 2).

**Table 2:** Chi-square comparison of offered and uptake rates of NHS Health Checks for male and female patients by age group

Age	Males						Females					
	%	ASR	Offered/Uptake	% Uptake Rate	CI	Sig	%	ASR	Offered/Uptake	% Uptake Rate	CI	Sig
40-44	25.6	-5.1	684/1345	0.51	(0.48–0.54)	***	24.0	-7.4	729/1727	0.42	(0.40–0.45)	***
45–49	20.8	-2.3	555/1551	0.36	(0.33–0.38)	*	21.3	2.1	645/1232	0.52	(0.50–0.55)	*
50–54	20.3	-1.0	543/1460	0.37	(0.34–0.40)	NS	17.5	-.3	532/1078	0.49	(0.46–0.52)	NS
55–59	13.4	-2.8	358/1040	0.34	(0.32–0.37)	**	11.9	-1.9	362/779	0.47	(0.43–0.50)	NS
60–64	10.1	7.0	270/512	0.53	(0.48–0.57)	***	11.6	2.0	351/658	0.53	(0.49–0.57)	*
65–69	6.7	10.9	178/250	0.71	(0.65–0.77)	***	9.4	5.3	285/463	0.62	(0.57–0.66)	***
70–74	3.1	6.1	81/120	0.68	(0.57–0.75)	***	4.0	7.8	130/162	0.80	(0.74–0.87)	***
Total	100 %		2669/6962				100 %		3034/6101			

***p < .001 **p < .01 *p < .05 NS *p* > .05

The findings revealed that there was significantly lowest uptake for males were found in patient’s aged 55–59 with an uptake rate of 0.34. Lowest uptake rates were predominantly found in the younger age groups including; ages 45–49, 50–54 and 40–44 with uptake rates of 0.36, 0.37 and 0.51 respectively. In contrast, higher uptake rates for males were found in the older age groups with highest uptake found for male patients aged 65–69 with an uptake rate of 0.71 (*p* < .001). Female patients showed a similar pattern to males with lower uptake rates found for the younger age groups with the significantly lowest uptake found for females aged 40–44 (p < .001). Highest uptake as found for males was found in the older age groups with the significantly highest uptake found for females aged 70–74 (*p* < .001).

### Ethnicity and gender

The ethnicity and gender of patients who were offered a Health Check was compared to actual uptake. Chi-square analysis revealed that uptake was not equally distributed across the total sample by ethnicity for both males (X^2^ = 1194.45, df = 15, *p <* .001) and females (X^2^ = 727.16, df = 15, *p <* .001) (Table 3).

**Table 3:** Chi-square comparison of offered and uptake rates of NHS Health Checks for eligible male patients

Ethnic Group	Males						Females					
	%	ASR	Offered/Uptake	% Uptake Rate	CI	Sig	%	ASR	Offered/Uptake	% Uptake Rate	CI	Sig
White: British	42.45	20.4	1133/1980	0.57	(0.55–0.59)	*	45.91	14	1393/2268	0.61	(0.59–0.63)	***
White: Irish	2.52	5.4	67/105	0.64	(0.54–0.73)	***	2.83	5	86/119	0.72	(0.64–0.80)	***
White: other	7.79	-7.0	208/774	0.27	(0.24–0.30)	***	8.0	-8.6	243/704	0.35	(0.31–0.38)	***
White/Black Caribbean	0.97	3.0	26/43	0.60	(0.46–0.74)	**	1.19	3.7	36/47	0.77	(0.63–0.87)	***
White/Black African	0.86	1.6	23/46	0.50	(0.36–0.64)	NS	0.92	2.8	28/39	0.72	(0.57–0.84)	**
White/Asian	0.37	3.6	10/11	0.91	(0.66–0.99)	***	0.16	-0.3	5/11	0.45	(0.19–0.74)	NS
Mixed: Other	0.45	0.5	12/28	0.43	(0.26–0.61)	NS	0.40	0.0	12/24	0.50	(0.31–0.69)	NS
Indian	4.76	6.8	127/208	0.61	(0.54–0.68)	***	5.70	8.0	173/228	0.76	(0.70–0.81)	***
Pakistani	3.33	1.3	89/209	0.43	(0.36–0.49)	NS	2.83	2.5	86/143	0.60	(0.52–0.68)	**
Bangladeshi	5.32	4.3	142/280	0.51	(0.45–0.57)	***	3.30	2.2	100/173	0.58	(0.50–0.65)	*
Asian: Other	2.10	4.0	56/97	0.58	(0.48–0.67)	***	1.38	-0.1	42/85	0.49	(0.39–0.60)	NS
Caribbean	4.80	8.7	128/185	0.69	(0.62–0.76)	***	4.88	6.2	148/209	0.71	(0.64–0.77)	***
African	4.65	-0.9	124/345	0.36	(0.31–0.41)	NS	4.65	-2.9	141/336	0.42	(0.37–0.47)	**
Black: Other	0.56	-0.8	15/46	0.33	(0.20–0.47)	NS	0.56	-0.5	17/37	0.46	(0.31–0.62)	NS
Chinese	0.52	2.7	14/21	0.67	(0.45–0.84)	**	0.43 %	3.2	13/14	0.93	(0.72–0.99)	***
Other	8.84	5.4	236/471	0.50	(0.46–0.55)	***	6.61 %	2.8	200/351	0.57	(0.52–0.62)	**
Not stated	8.54	-29.5	259/2113	0.12	(0.11–0.14)	***	2.21 %	-21.3	311/1313	0.24	(0.21–0.26)	***
Total	100 %		2669/6962				100 %		3034/6101			

***p < .001 **p < .01 *p < .05 NS *p* > .05

Chi-square analysis revealed that for males ‘Black Caribbean had the highest uptake rate across all ethnic groups with an overall uptake rate of 0.69 (*p* < .001). Findings also revealed that both Asian Indian (*p* < .001) and ‘White British’ (*p* < .01) patients also had a significantly higher uptake rate compared to other ethnic groups with an uptake rate of 0.61 and 0.57 respectively. The lowest overall uptake of the NHS Health Checks was found for male patients categorised as ‘Any Other White’ background with an overall uptake rate of 0.27 (*p* < .001), closely followed by ‘Any other Black Background’ and ‘Black African with an uptake rate of 0.33 and 0.36 (*p* < .001).

The findings revealed that the significantly highest overall uptake of the NHS Health Check for females was ‘Asian Indian’ (*p* < .001) who had an overall uptake rate of 0.76. This was closely followed by ‘Black Caribbean (*p* < .001) and ‘White British’ (*p* < .001) female patients with uptake rates of 0.71 and 0.61 respectively. In contrast, the significantly lowest overall uptake rate was found for ‘Any Other White’ (*p* < .001) and ‘Black African’ (*p* < .01), and ‘Mixed White and Asian’ patients who had an overall uptake rate of 0.35 and 0.42 respectively.

### The impact of ethnicity and gender on invitation method

Invitation data was recorded for a total 12,048 of the total 13,063 NHS patients across the 30 Luton practices. Analysis confirmed that of those recorded as invited there was an overall uptake rate of 32.7 % (*N* = 3,938/12,048). Findings further revealed that uptake varied across recruitment method. For example, highest uptake rate was found for verbal face to face with an uptake rate of 71.9 % with uptake rate by telephone (43 %) and letter (29.5 %) invitation markedly lower.

The ethnicity and gender of patients who were offered a Health Check by invitation method was compared to actual uptake. Chi-square analysis uncovered that uptake by invitation method was not equally distributed across the total sample across ethnicity and gender when invited by an ‘invitation letter’ (males: X^2^ = 149.122, df = 15, *p <* .001; females: X^2^ = 356.203, df = 15, *p <* .001), ‘verbally face-to-face’ invitation (males: X^2^ = 82.243, df = 15, *p <* .001; females: X^2^ = 30.410, df = 15, *p <* .001) alongside ‘telephone invitation’ (males: X^2^ = 51.614, df = 15, *p <* .001; X^2^ = 124.661, df = 15, *p <* .001).

### Invitation letter

Chi-square analysis indicated that for males ‘Mixed White and Asian were significantly more likely to uptake an NHS Health Check after being invited by an invitation letter, with findings revealing an uptake rate of 0.70 (*p* < .01). However, male patients categorised as ‘Any Other White Background’ were shown to have the significantly lowest uptake when invited by letter, with an uptake rate of 0.19 (*p* < .001) (Table 4).

**Table 4:** Chi-square comparison of offered and uptake rates of NHS Health Checks by letter invitation method for male and female patients by ethnicity

Ethnic Group	Males						Females					
	%	ASR	Offered/Uptake	% Uptake Rate	CI	Sig	%	ASR	Offered/Uptake	% Uptake Rate	CI	Sig
White: British	43.78	7.7	672/1853	0.36	(0.34–0.39)	***	47.72 %	10.5	794/2112	0.38	(0.36–0.40)	***
White: Irish	2.48	2.3	38/94	0.40	(0.31–0.51)	*	3.13 %	4.3	52/109	0.48	(0.39–0.57)	***
White: other	8.99	-6.5	138/713	0.19	(0.27–0.22)	***	8.53 %	-4.4	142/649	0.22	(0.19–0.25)	***
White/Black Caribbean	1.30	2.7	20/41	0.49	(0.34–0.64)	**	1.14 %	2.3	19/42	0.45	(0.31–0.60)	*
White/Black African	0.78	-0.2	12/42	0.29	(0.17–0.43)	NS	0.90 %	1.8	15/35	0.43	(0.27–0.59)	NS
White/Asian	0.46	2.8	7/10	0.70	(0.40–0.92)	**	0.24 %	0.7	4/10	0.40	(0.15–0.70)	NS
Mixed: Other	0.39	-0.7	6/26	0.23	(0.10–0.41)	NS	0.06 %	-2.4	1/20	0.05	(0.00–0.20)	*
Indian	4.89	2.6	75/197	0.38	(0.32–0.45)	**	5.35 %	4.7	89/201	0.44	(0.38–0.51)	***
Pakistani	3.13	-1.9	48/201	0.24	(0.18–0.30)	NS	1.86 %	-2.3	31/149	0.21	(0.15–0.28)	*
Bangladeshi	5.02	0.7	77/242	0.32	(0.26–0.38)	NS	3.37 %	2.3	56/148	0.38	(0.30–0.46)	*
Asian: Other	1.63	-0.3	25/88	0.28	(0.20–0.38)	NS	1.44 %	0.3	24/78	0.31	(0.20–0.40)	NS
Caribbean	4.30	2.4	66/174	0.38	(0.31–0.45)	*	5.41 %	5.1	90/197	0.46	(0.39–0.53)	***
African	4.82	-2.8	74/324	0.23	(0.19–0.28)	**	4.45 %	-2.4	74/316	0.23	(0.19–0.28)	*
Black: Other	0.59	-1.1	9/41	0.22	(0.11–0.36)	NS	0.60 %	0.0	10/34	0.29	(0.16–0.46)	NS
Chinese	0.52	1.2	8/19	0.42	(0.22–0.64)	NS	0.48 %	2.6	8/13	0.62	(0.35–0.84)	**
Other	7.74	-0.3	119/409	0.29	(0.29–0.25)	NS	5.89 %	0.6	98/319	0.31	(0.26–0.36)	NS
Not stated	9.18	-5.7	141/687	0.07	(0.06–0.08)	***	9.43 %	-14.6	157/1243	0.13	(0.11–0.15)	***
Total	100		1535/ 5161				100 %		1664/5675			

***p < .001 **p < .01 *p < .05 NS *p* > .05

Female patients who had the highest NHS Health Check uptake rate by the letter invitation were Chinese with an uptake rate of (0.62) (*p* < .01). The second and third highest uptake rates were found for ‘White Irish’ (*p* < .001) and ‘Black African’ (*p* < .05) with uptake rates of 0.48 and 0.46 respectively. The lowest group to uptake rate based on an invitation letter was ‘Any Other Mixed Background’ who had an uptake rate of 0.05 (*p* < .05). The second and third lowest user groups were ‘Asian Pakistani’ (*p* < .05) and ‘White Other’ (*p* < .001) who had an uptake rate of 0.22 and 0.22 respectively.

### Verbal face-to-face Invitation

For males the results showed that the significantly highest uptake rate for the NHS Health Check compared to the other ethnic groups by verbal invitation for males was ‘White British’ patients with an uptake rate of 0.72 (*p* < .001). Lowest uptake was found for ‘Bangladeshi’ (*p* < .001) and ‘Pakistani’ (*p* < .05) male patients who had an uptake rate of 0.43 and 0.47 respectively. For females, ‘White Irish’ (*p* < .05) and ‘White British’ (*p* < .001) patients were revealed to have the significantly highest uptake rates with uptake rates of 0.93 and 0.79 respectively. Lower than expected uptake rates for ‘face to face’ invitations were not found across all ethnic groups for females(Table 5).

**Table 5:** Chi-square comparison of offered and uptake rates of NHS Health Checks by verbal face-to-face invitation method for male and female patients by ethnicity

Ethnic Group	Males						Females					
	%	ASR	Offered/Uptake	% Uptake Rate	CI	Sig	%	ASR	Offered/Uptake	% Uptake Rate	CI	Sig
White: British	44.36	7.7	114/158	0.72	(0.70–0.82)	***	44.1	3.2	127/161	0.79	(0.72–0.85)	***
White: Irish	3.50	1.2	9/10	0.90	(0.63–0.99)	NS	4.86	2.0	14/15	0.93	(0.74–0.99)	*
White: other	7.00	-0.7	18/27	0.67	(0.48–0.82)	NS	5.90	-1.9	17/31	0.55	(0.38–0.71)	NS
White/Black Caribbean	1.56	0.4	4/5	0.80	(0.37–0.99)	NS	3.13	0.0	9/13	0.69	(0.42–0.89)	NS
White/Black African	2.72	0.3	7/9	0.78	(0.46–0.96)	NS	2.43	1.1	7/8	0.88	(0.56–0.99)	NS
White/Asian	0	-1.6	0	0	0	NS	0.35	-0.6	1/1	1.00	(0.15–1.0)	NS
Mixed: Other	0.39	-0.7	1/1	1.0	(0.15–1.0)	NS	1.04	0.2	3/4	0.75	(0.28–0.98)	NS
Indian	5.84	0.6	15/19	0.79	(0.58–0.92)	NS	3.47	-1.7	10/19	0.53	(0.31–0.74)	NS
Pakistani	2.72	-2.3	7/15	0.47	(0.24–0.71)	*	1.39	-0.7	4/7	0.57	(0.23–0.87)	NS
Bangladeshi	3.50	-3.2	9/21	0.43	(0.23–0.64)	***	2.08	-1.1	6/11	0.55	(0.27–0.81)	NS
Asian: Other	2.33	-0.4	6/9	0.67	(0.35–0.91)	NS	1.04	0.2	3/4	0.75	(0.28–0.98)	NS
Caribbean	4.28	0.5	11/14	0.79	(0.53–0.94)	NS	4.51	-0.1	13/19	0.68	(0.46–0.86)	NS
African	2.72	-0.2	7/10	0.70	(0.39–0.92)	NS	4.86	0.8	14/18	0.78	(0.56–0.93)	NS
Black: Other	1.18	0.1	3/4	0.75	(0.28–0.98)	NS	0.69	-0.1	2/2	1.00	(0.38–1.0)	NS
Chinese	0.39	0.7	1/1	1.0	(0.15–1.0)	NS	0.69	-0.6	2/2	1.00	(0.38–1.0)	NS
Other	12.06	-3.0	31/55	0.56	(0.43–0.69)	***	9.03	0.3	26/36	0.72	(0.56–0.85)	NS
Not stated	5.45	-4.6	14/35	0.40	(0.25–0.57)	***	10.43	-3.2	30/58	0.52	(0.39–0.64)	***
Total	100 %		257/393				100 %		288/ 409			

***p < .001 **p < .01 *p < .05 NS *p* > .05

### Telephone Invitation

Telephone invitations were the least used invitation method with small sample sizes across some ethnic groups (Table 6). The results nonetheless suggested that for males ‘White other’ patients had the significantly lowest uptake rate across all ethnic groups with an uptake rates of 0.10 (*p* < .001). ‘Pakistani’ and ‘Bangladeshi’ male patients were found to have the significantly highest uptake rates across all ethnic groups when invited by telephone with an uptake rate of 1.0 and 0.58 respectively (*p* < .01).

**Table 6:** Chi-square comparison of offered and uptake rates of NHS Health Checks by telephone invitation method for male and female patients by ethnicity

Ethnic Group	Males						Females					
	%	ASR	Offered/Uptake	% Uptake Rate	CI	Sig	%	ASR	Offered/Uptake	% Uptake Rate	CI	Sig
White: British	16.22	-1.8	18/78	0.23	(0.15–0.33)	NS	23.76	-5.4	0/32	0.00	(0.00–0.01)	***
White: Irish	1.81	-0.4	2/8	0.25	(0.05–0.59)	NS	1.98	5.7	24/25	0.96	(0.84–0.99)	***
White: other	4.50	-3.5	5/49	0.10	(0.04–0.21)	***	9.90	-4.0	2/26	0.08	(0.01–0.22)	***
White/Black Caribbean	0.90	0.6	0	0	-		0	3.7	10/10	1.00	(0.83–1.00)	***
White/Black African	0.90	0.6	1/1	1.00	(0.15–1.0)	NS	0	0	0	-	-	-
White/Asian	0.90	0.6	0	0	-		0	1.1	1/1	1.00	(0.15–1.00)	NS
Mixed: Other	1.81	1.3	2/3	0.67	(0.16–0.98)	NS	0	0	0	-	-	-
Indian	2.70	0.6	3/7	0.43	(0.13–0.77)	NS	9.90	-1.5	0/3	0.00	(0.00–0.00)	NS
Pakistani	1.81	2.6	3/3	1.00	(0.38–1.0)	**	5.05	3.7	10/10	1.00	(0.83–1.00)	***
Bangladeshi	14.41	2.7	16/30	0.53	(0.36–0.70)	**	15.84	-.01	5/12	0.42	(0.15 – 0.72)	NS
Asian: Other	0.90	-1.2	1/8	0.13	(0.01–0.45)	NS	0	3.2	16/21	0.76	(0.53–0.91)	***
Caribbean	3.60	0.8	4/9	0.44	(0.17–0.75)	NS	0.99	-1.5	0/3	0.00	(0.00–0.00)	NS
African	2.70	-0.7	3/13	0.23	(0.06–0.50)	NS	2.97	-0.04	1/3	0.33	(0.02–0.83)	NS
Black: Other	0.90	-0.6	0/4	0.00	(0.62–1.0)	NS	2.02	2.0	3/3	1.00	(0.83–1.00)	*
Chinese	0.90	0.6	1/1	1.00	(0.15–1.0)	NS	0	1.6	2/2	1.00	(0.83–1.00)	NS
Other	23.42	4.0	26/45	0.58	(0.43–0.72)	***	10.89	-3.3	0/13	0.00	(0.00–0.00)	***
Not stated	21.62	-0.8	24/85	0.28	(0.19–0.38)	NS	15.84	-1.0	11/31	0.36	(0.20–0.53)	NS
Total	100		109/ 344				100		85/195			

***p < .001 **p < .01 *p < .05 NS *p* > .05

For females, the findings revealed that ‘Mixed White and Black Caribbean’, ‘Pakistani’ ‘Irish’ ‘Asian Other’ females who were invited by telephone had a significantly higher uptake compared to the other ethnic groups with reported uptake rates of 1.0, 1.0, 0.96 and 0.76 respectively (*p* < .001). However, female patients categorised as ‘White British’ ‘Any Other White Background’ and ‘Other not stated’ showed the significantly lowest uptake rate across all ethnic groups when invited by telephone with uptake rates of 0.0, 0.08 and 0.36 (*p* < .001).

## Discussion

### Main findings of this study

A key finding of the present research study was that the overall uptake rate for Luton was 44 %, which is markedly lower than the Department of Health’s projected set target of 50–75 % [9] and the national average (47.95) [18]. However, uptake has been found to be varied, for example Stoke-on-Trent found an uptake rate of the NHS Health Check programme of 61.6 % which whilst it varied by GP practice it still demonstrated a markedly higher uptake rate than the national average [7]. Studies which have also explored uptake in culturally diverse settings have also found low uptake rates [6] which suggests the variations in uptake may reflect the wider socio-demographic characteristics of the local authority population.

When accounting for gender, females marginally met the target uptake rate (50 %), although uptake by males (38 %) was significantly lower (*p* < .001). Variation in uptake by gender is supported by another research study purporting female status as being predictive of higher NHS Health Check uptake [7]. There were also age differences found with findings suggesting that there was a markedly lower uptake of the Health Check programme in the younger age groups with higher uptake in the older age groups (65 years and older) for both males and females. This is not a new finding in that previous studies have also found that higher age groups are more likely to have an NHS Health Check [6] however what remains less clear is why this variation exists.

The results indicated that those who were from the most deprived wards had a significantly lower uptake, which contradicts previous research [19] and ultimately suggest that NHS Health Checks are not reaching patients who are in the most deprived wards in Luton. Low socioeconomic status and lack of uptake of healthcare services is a well-defined link in academic literature [20, 21] and engaging the poorer sections of the communities who often have the highest rates of morbidity and mortality should remain a key focus for engagement. Exploring ‘patient’ views on the NHS Health Check programme should be a key priority. This will enable a deeper understanding of the barriers to uptake and to determine the usefulness of this programme and related incentives in engaging these sections of the community.

In relation to ethnicity findings revealed that ‘White British’ patients were significantly more likely to uptake an NHS Health Check for both males and females patient groups. Similarly, across both gender groups, overall it was found ‘Black Caribbean’ and ‘Indian’ patients also had a significantly higher uptake rate. However, both male and female patient groups from ‘Any Other White Background’ and ‘Black African’ had a significantly lower uptake of an NHS Health Check compared to all ethnic groups. This result contradicts previous findings which have suggested that there has been no ethnic variations to uptake [6, 19].

This research then explored the successfulness of invitation approach on uptake. It was clear that face-to-face invitations held the most success with an overall uptake rate of 71.9 % with uptake rates for both telephone (43 %) and letter (29.5 %) invitations markedly lower. Moreover, there was a variation of uptake across ethnicity by invitation method. Invitation letter was the most common form of invitation with 12,209 letters sent to eligible patients. This method was most effective for ‘Mixed White and Asian’ male and ‘Chinese’, ‘Irish’ and ‘African’ female patients. However, was least successful for ‘Any Other White Background’ and ‘Pakistani’ female patients who revealed to have lowest NHS Health Check uptake rates.

A face-to-face invitation was delivered to 801 eligible patients. This method was most effective for ‘White British’ male and female patients. However, findings suggested that verbal invitation was the least effective method for inviting ‘Bangladeshi’ and ‘Pakistani’ males. Finally, invitation by telephone was the least common method with 210 patients being invited to an NHS Health Check using this approach. However, where this method was used, it was most effective for Asian (Bangladeshi, Pakistani, Asian Other) patients but least effective for ‘White British’ and ‘Any Other White Background’ patients.

Ethnicity and gender appeared to play a key role in determining response to different forms of communication used to invite the patient to the NHS Health Check. There was wide variation across both ethnic and gender groups, however the groups that were responding least to the invitations were: ‘Any Other White Background’, in particular females from this ethnic group and males from ‘Asian Other’ and ‘Black African’ ethnic groups. There has been no research, which has explored factors effecting uptake by ethnicity, and invitation method, which may or may not impact uptake but preliminary evidence has highlighted that GPs from similar ethnic backgrounds can improve usage [5, 22].

The ‘Any Other White Background’ ethnic group in Luton is assumed to be predominantly Polish due to the influx of Polish migrants to Luton since 2004 [23]. Although the Polish population register with a UK GP practice, some research has highlighted that whilst in the UK, there is a preference to travel back to Poland to access healthcare services [23–25]. Other barriers to NHS Health Check uptake could relate to problems registering with primary care services, cultural barriers and language and interpretation problems. [25] For example, many Polish migrants do not speak English proficiently [26] and with no standard method of invitation across all GP practices, it is not clear if invitations made are offered in non-English languages such as Polish. Moreover, whilst the NHS Health Check website does provide materials in Polish, there is no evidence of how effective these are in reaching the community. In essence, there is a need to improve health literacy [27] about the NHS Health Check as Polish patients may misunderstand what the programme is for or what the patient needs to do to book an appointment.

In addition, Polish migrants have been reported to have some misunderstandings of the NHS and hold a lack of trust in the UK health service [28]. Literature on the relationship between the differences in accessing healthcare in the UK and Poland shows that Polish patients have better access without referral to specialist doctors than in the UK [29]. Therefore, Polish patients may prefer to see a specialist conduct the NHS Health Check as opposed to a health care assistant or nurse. In addition, the NHS Health Check puts the onus onto the patient to call their GP practice to make their appointment to have the check. This process may be deterring Polish patients from accessing the service, particularly if the patient needs to call to discuss this and may not feel confident speaking English or may not understand that this is the first step in having an NHS Health Check [26].

### Limitations of this study

There are a number of limitations that should be acknowledged. Firstly, the data did not include invitation methods prior to the start date (1^st^ April, 2013), therefore, patients may have been invited prior to the baseline and been recorded as having a Health Check during the study period. Moreover, invitations may have been made during the study period but the NHS Health Check uptake may have occurred outside of the study period i.e. after 31^st^ March 2014. Whilst it is not possible to know the true number of cases this relates to the uptake of the NHS Health check following an invitation often follows shortly after therefore, it is likely that this issue only related to a small number of cases. The ‘White Other’ which is assumed to consist of East European patients is in many ways different from the other groups where some caution should be taken. However, analysis was re run with this group excluded and no systematic differences were found.

There were a number of patients where their ethnicity was unknown (Males *n* = 2,113; Females *n* = 1,313). Whilst ethnicity should be recorded on the GP systems, a number of GP practices do not routinely update or audit their data to check that this has been done. There was also a discrepancy between those recorded in being invited through the invitation methods and actual NHS Health Check uptake (*N* = 1,747). This could be because it has not been recorded, and/or the NHS Health Check had been conducted at the GP practice ‘on-the-spot’ when they have an appointment for an ailment, rather than having been invited. Third party opportunistic invitation, which was not included in analysis, may have also impacted on this discrepancy. While these potential factors may have an influence on overall rates, there is no reason to expect response rates of particular groups would be differentially affected. Moreover CCG’s should take active steps in ensuring that GP practice data is both accurate and reliable to maintain the integrity of research, which will contribute to future decision planning.

A limitation that should be considered is that there is no standardised invitation process across the GP practices. This makes it difficult to determine the approach used to recruit participants and to be able to compare by practice. However, this in itself highlights the need to develop a tailored recruitment protocol for local authorities and GP practices to provide a more transparent and consistent approach. Finally, whilst the presented findings provide an indication of how the invitation process may be influenced by gender and ethnicity it would be useful to confirm these findings through the delivery of a randomised controlled trial where participants could be randomly allocated to the different contact methods.

## Conclusions

NHS Health Checks is a national prevention programme, which aims to assess the risk of all 40–74 year olds in England of heart disease, stroke, diabetes, kidney disease and certain types of dementia. Through the identification of risk, patients are supported by local services to reduce or manage that risk to prevent the onset of preventable disease and subsequently reduce morbidly and mortality [1].

A core focus of the programme is to reduce health inequalities by increasing access. Therefore, there is a clear importance to understanding how to engage culturally diverse populations in the UK in NHS Health Checks [20]. Whilst research has suggested that patients from South Asian and mixed ethnic backgrounds had higher uptake compared with White British [6] this finding has not been consistent [5].

The present study shows that ethnicity, gender and method of invitation can illustrate how different patient groups are engaged in different ways. This is an area that needs further attention as thus far, there has been no research which has explored ethnicity and gender in relation to invitation method used to recruit patients [8] which remains a key priority for the NHS Health Checks research strategy [4]. As such, the present research study aimed to evaluate the variation of ethnicity and gender by invitation method on the uptake of the NHS Health Checks programme focusing on Luton, a culturally diverse urban town within England.

The findings revealed that highest uptake rate of the NHS Health Check were White British patients, with the lowest uptake found ‘Any Other White Background’ patients, who could be assumed to be Eastern European patients, in particular Polish patients due to the influx over the past decade. The analysis also highlighted that different invitation methods were effective for different ethnic and gender groups, for example, the invitation letter appealed most to the ‘White British’ population and the verbal invitation encouraged significantly more ‘Asian’ patients to uptake an NHS Health Check.

The presented research suggests that a ‘one size fits all’ approach to recruitment may not be the best approach. Instead it is suggested that established protocols of invitation are specifically designed for maximizing the response rate for each population group. Tailoring the invitation methods and ensuring they are culturally sensitive could well make a positive contribution to increasing uptake of the NHS Health Check programmes [30]. However, future research is needed, specifically the development and delivery of a RCT to investigate how the invitation method impacts on the NHS Health Checks to confirm the findings presented. This evidence base will inform if there is a need for a more tailored approach to recruitment to improve uptake, and if so, a consideration should be made to the potential usefulness of a nationally consistent method. Future research should also aim to capture the facilitators and barriers which impact on the uptake of the NHS Health Check programme, such as deprived culturally diverse population groups to explore why uptake rates are low among these groups and how public health teams can better engage with these communities.

## Footnotes

1 Participants will be referred to as ‘patients’ in this study as they were recruited from GP practices.
